# Influence of kiwifruit on gastric and duodenal inflammation-related gene expression in aspirin-induced gastric mucosal damage in rats

**DOI:** 10.1038/s41598-020-70006-0

**Published:** 2020-08-03

**Authors:** Kerry L. Bentley-Hewitt, Matthew Perrott, Christine A. Butts, Duncan I. Hedderley, Halina M. Stoklosinski, Shanthi G. Parkar

**Affiliations:** 1 The New Zealand Institute for Plant and Food Research Limited, Private Bag 11600, Palmerston North, 4442 New Zealand; 2 School of Veterinary Science, Massey University, Palmerston North, New Zealand

**Keywords:** Gastrointestinal models, Circadian rhythms, Immunology

## Abstract

Kiwifruit (KF) contains bioactive compounds with potential anti-inflammatory properties. In this study, we investigated the protective effects of KF on gastric and duodenal damage induced by soluble aspirin in healthy rats. Sixty-four male Sprague Dawley rats were allocated to eight experimental treatments (n = 8) and the experimental diets were fed for 14 days ad libitum. The experimental diets were 20% fresh pureed KF (green-fleshed and gold-fleshed) or 10% glucose solution (control diet). A positive anti-inflammatory control treatment (ranitidine) was included. At the end of the 14-day feeding period, the rats were fasted overnight, and the following morning soluble aspirin (400 mg/kg aspirin) or water (control) was administered by oral gavage. Four hours after aspirin administration, the rats were euthanized and samples taken for analysis. We observed no significant ulcer formation or increase in infiltration of the gastric mucosal inflammatory cells in the rats with the aspirin treatment. Despite this, there were significant changes in gene expression, such as in the duodenum of aspirin-treated rats fed green KF where there was increased expression of inflammation-related genes *NOS2* and *TNF-alpha*. We also observed that gold and green KF diets had a number of contrasting effects on genes related to inflammation and gastro-protective effects.

## Introduction

Kiwifruit (KF) are linked to multiple health benefits^1^. KF contain bioactives (e.g. polyphenols and galactolipids) that have been shown to reduce inflammation^2,3^. For example, monogalactosyl diacylglycerol (MGDG), a group of galactolipids, some of which are present in KF, resulted in an anti-inflammatory response in a mouse footpad model of inflammation^4^. We have previously established 20 µM of KF galactolipids can suppress an inflammatory signaling protein (IL-2) in antigen-stimulated peripheral blood mononuclear cells (PBMC) cultured in vitro and concentrations exceeding 20 µM of these galactolipids reach the stomach and duodenum of rats fed KF (Bentley-Hewitt, unpublished). Therefore, bioactive concentrations of galactolipids could interact with infiltrating immune cells or resident immune cells in the stomach or duodenum. Polyphenols also have well-established protective and therapeutic potential in reducing peptic ulcers, one mechanism of which involves suppression of oxidative mucosal damage^5^. In addition, KF is a good source of vitamin C, with green-fleshed containing approximately 93 mg/100 g and gold-fleshed 161 mg/100 g^1^. Vitamin C has been shown to prevent gastric damage (960 mg or approximately six gold-fleshed KF) when given with aspirin (acetylsalicylic acid)^6^. Furthermore, pectin, a soluble fibre present in KF, was found to prevent non-steroidal anti-inflammatory drug (NSAID)-induced lesion formation, along with other soluble fibers^7^.

NSAIDs, such as aspirin and indomethacin, are commonly used to relieve pain, fever and inflammation. Aspirin use has been associated with potentially serious dose-dependent gastrointestinal complications, including gastrointestinal ulceration and localized mucosal inflammation^8–10^. Aspirin leads to inflammation by activating macrophages to produce cytokines that then promote infiltration of neutrophils^11^. Cytokines such as tumor necrosis factor-alpha (TNF-alpha), interleukin (IL)-6 and IL-10 are involved in acute-phase inflammation as well as in maintenance, regulation and severity of gastric ulceration^12^.

Inflammatory signaling is complex and involves interaction with genes that have a range of functions. This includes genes involved in serotonin (a major tryptophan metabolite) synthesis (e.g. *tryptophan hydroxylase*, *TPH*) and transport (e.g. *solute carrier family 6 member 4*, *SLC6A4*), since serotonin in the gastrointestinal tract induces inflammation^13–15^. Similarly, genes encoding tight junction proteins (e.g. *claudins 1* and *4*, *CLDN1/4* and *occludin*, *OCLN*), which contribute to a vital defensive barrier in the gut, have a critical role in controlling inflammatory response^16^. Most recently, research has established that inflammatory signaling is under circadian control, via a set of core clock genes (e.g. *Circadian Locomotor Output Cycles Kaput*, *CLOCK* and *period 1*, *2* and *3*, *PER 1/2/3*^17^.

We hypothesized that consuming green KF (*Actinidia chinensis var. deliciosa* ‘Hayward’, marketed as Zespri Green Kiwifruit) and gold KF (*Actinidia chinensis* var. *chinensis* ‘Zesy002′ marketed as Zespri SunGold Kiwifruit) reduces gastric and duodenal mucosal inflammation caused by the intake of NSAIDs. In the present study, we investigated the effects of a diet rich in KF on aspirin-induced gut inflammation in healthy rats by assessing gastric and duodenal mucosa injury and expression of genes involved in inflammation and gastrointestinal function.

## Results

### Gastric inflammation using histological analysis

**Figure 1:**
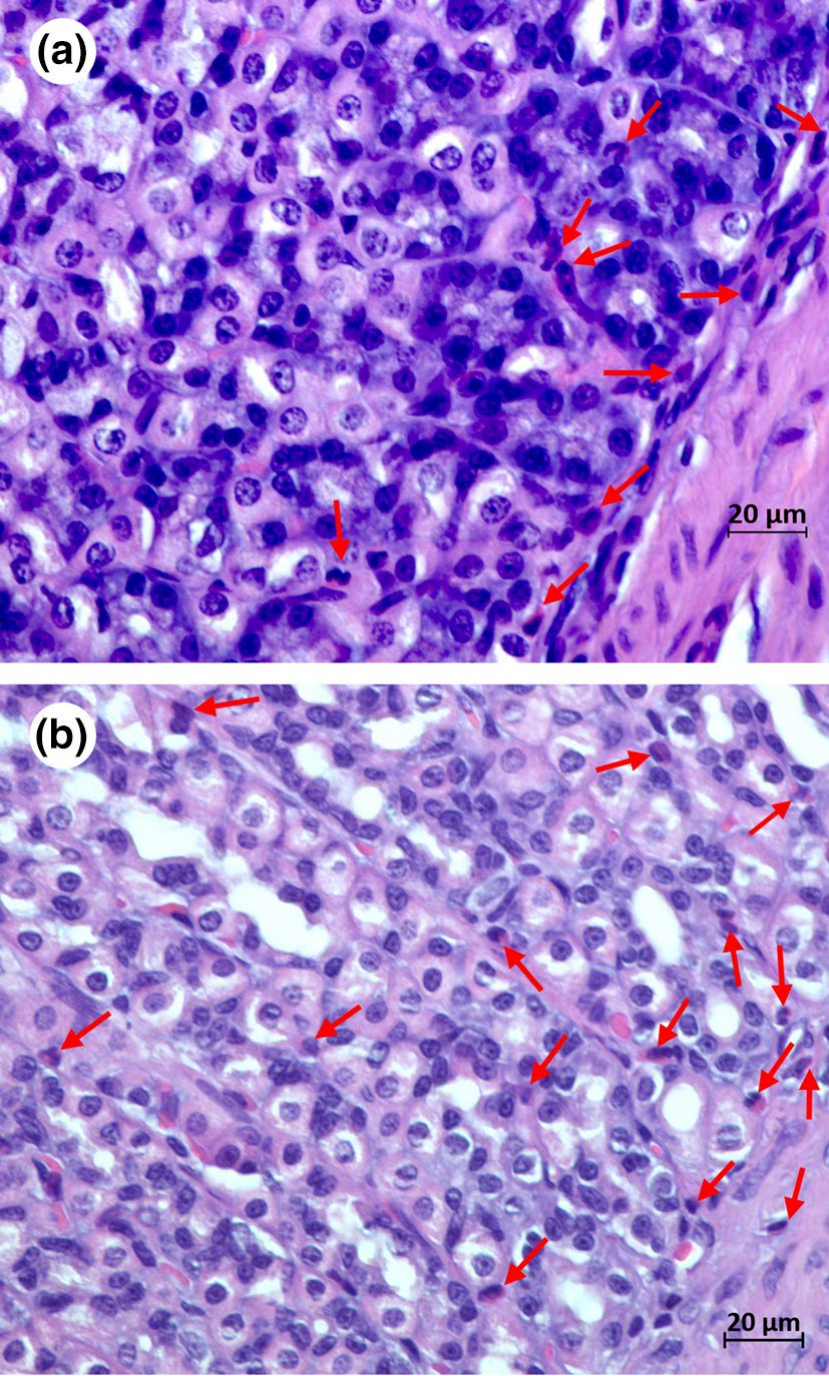
Example images from haematoxylin and eosin stained stomach tissue slides taken on a Zeiss Axio Imager Z2 microscope (objective × 40) from (**a**) rat 11 fed green kiwifruit with no aspirin treatment, and (**b**) rat 41 fed green kiwifruit with aspirin treatment. Inflammatory cells are identified are marked with a red arrow.

Few ulcers were observed in the stomach and duodenal tissues after the aspirin challenge. Two of the 64 rats had visible duodenal ulcers < 1 mm^2^ (1 green KF, no aspirin; 1 positive control drug, with aspirin), and in nine rats across the experimental groups we observed gastric ulcers < 1 mm^2^ with one of these having larger ulcers (1–3 mm^2^). There was a significant effect of diet (*p* = 0.044) on the number of gastric ulcers, with the green KF diet producing less than the gold KF or positive control diets (Table 1). The number of inflammatory cells in stomach tissue at magnification (× 40) are presented in Supplementary Fig. 1. Examples of the scored images are shown in Fig. 1. We did not observe any significant differences in inflammatory cell number following administration of aspirin or the experimental dietary treatments. The model used was adapted from a previous model used in-house^18^, which did produce visible small ulcers.

**Table 1:** Gastric ulcer scores.

Treatment	Rat number	Mean	SEM
Control with aspirin	8	0.375^abc^	0.375
Gold KF with aspirin	8	0.750^bc^	0.750
Green KF with aspirin	8	0.000^a^	0.000
Control + ranitidine with aspirin	8	0.250^abc^	0.164
Control without aspirin	8	0.000^a^	0.000
Gold KF without aspirin	8	0.500^abc^	0.327
Green KF without aspirin	8	0.125^ab^	0.125
Control + ranitidine without aspirin	8	1.000^c^	0.756

Source of variation	Degrees of freedom	Deviance ratio	*p* value
Treatment_group	3	2.9	**0.044**
Aspirin_treatment	1	0.1	0.738
Treatment group.Aspirin	3	2.2	0.101
Residual	56		

Mean values with a different letter differ significantly. *KF* kiwifruit, *SEM* Standard error of the mean.

Although we were unable to demonstrate pro-inflammatory changes at the histological level, we were able to establish a new scoring methodology for histological analysis. We determined that seven images per rat (n = 8 rats per treatment) were sufficient to show a difference in the inflammatory cell count, the mean inflammatory cell count for each animal was plotted based on 2, 3, 4, 5, 6 or 7 images per rat. Calculating coefficients of variability (standard deviation/mean × 100%) for each rat, the total inflammatory cells ranged from 22 to 166% (median 48%, interquartile range 37% to 60%). This suggests that to achieve a mean (inflammatory cell count) per animal that is within 50% of the true mean (i.e. standard error = 25%) for most (75%) animals, we would need data from a minimum of six slides.

Food intake and weight gain during the feeding period were not significantly different between the experimental groups (Supplementary Table S3).

### Principal Component Analysis

**Figure 2:**
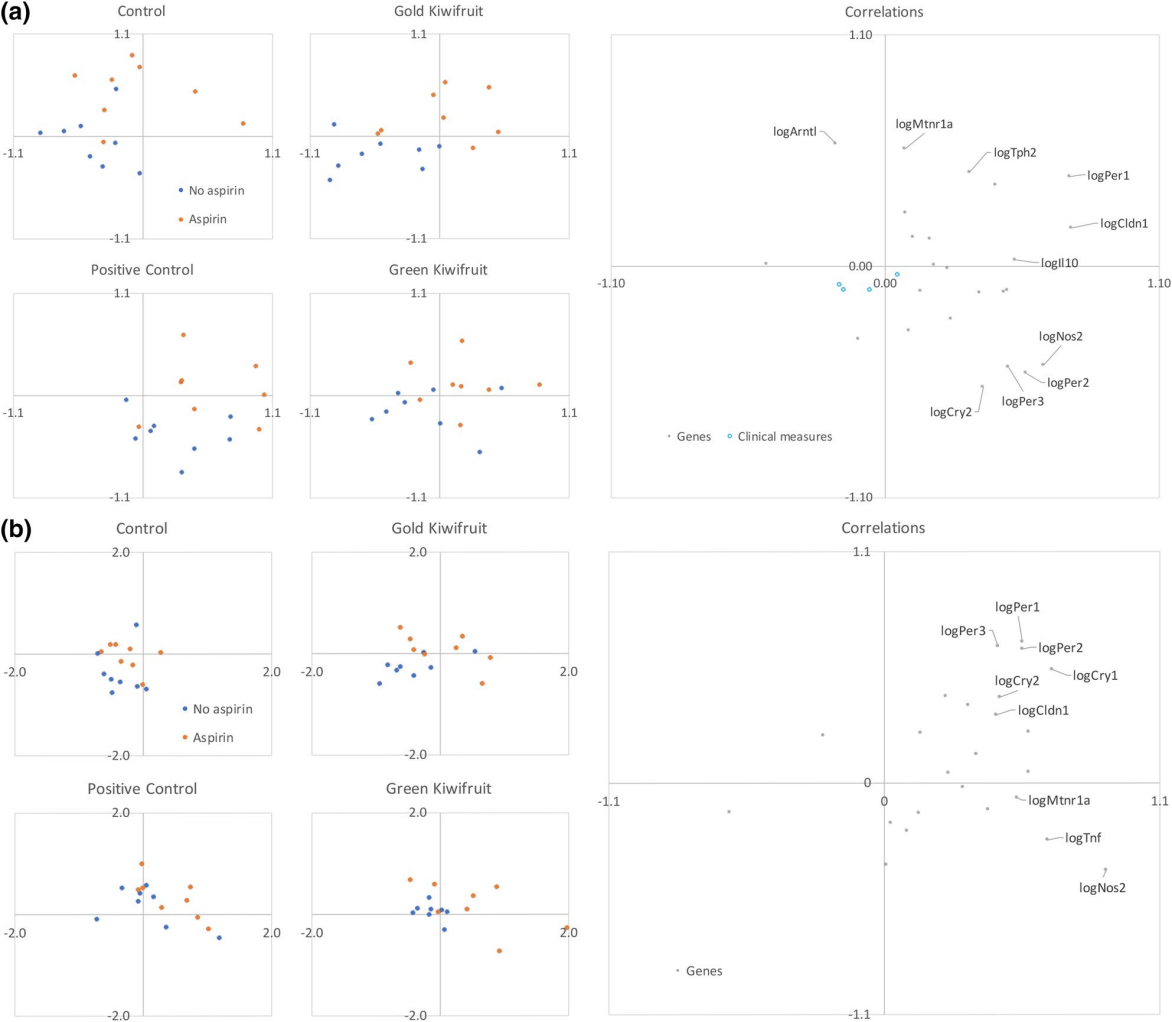
A principal component analysis using log-transformed gene expression in a) stomach and b) duodenal tissue. Results from each animal plotted separately, color coded by aspirin/no aspirin. For the stomach, principal components 1 (horizontal) and 2 (vertical accounted for 25% and 14% of variance, respectively; for the duodenum, 35% and 18%, respectively. For the stomach, four clinical measures (number of inflammatory cells in submucosa and mucosa, total number inflammatory cells, gastric ulcer score, shown with blue circles) were also correlated with the principal components.

Using all measures, including genes, inflammatory cell counts and ulcer scores, and plotting the first two principal components, subdivided by diet and aspirin or not, the aspirin and diet effects appeared to be separate and differ between tissue types (Fig. 2). The PCA showed that in the stomach, green KF and ranitidine appeared higher on PC1 compared with aspirin treatment, which was higher on PC2 (Fig. 2a). For the stomach, the higher values on PC1 indicated increases in *IL-10*, *CLDN1*, *NOS2, PER1, PER2, PER3*; higher values on PC2 indicated increases in *ARNTL, MTNR1A*, *TPH2* and decreases in *CRY2*. Whereas in the duodenum, aspirin given with green KF and ranitidine increased for both PC1 and PC2 (Fig. 2b). For the duodenum, the higher values on PC1 indicated increases in *CRY1, CRY2, CLDN1, MTNR1A, TNF-alpha* and *NOS2*; higher values on PC2 indicated increases in *PER1, PER2, PER3.*

### Gene expression

**Figure 3:**
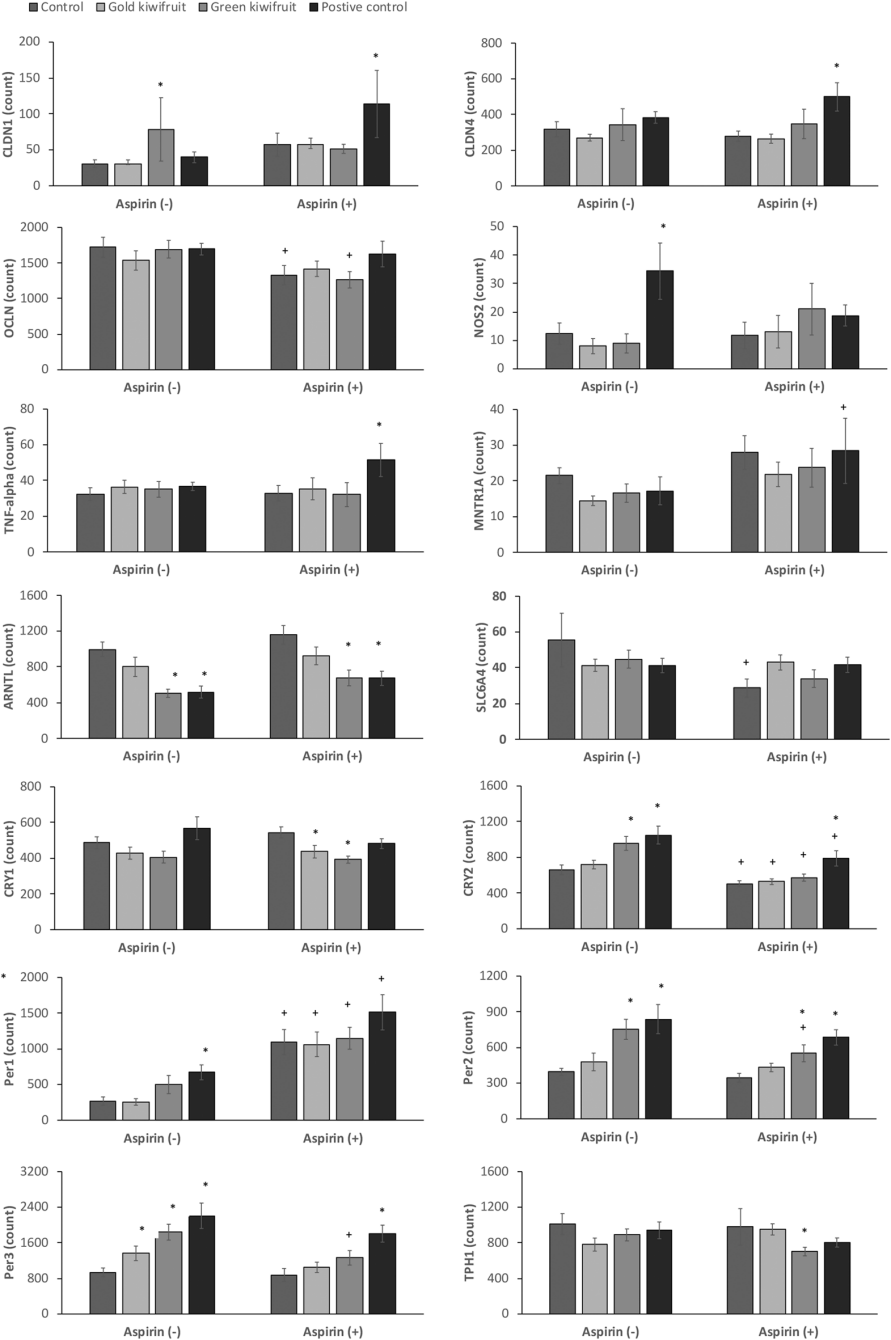
Stomach gene expression counts normalized to reference genes (n = 8) from rats fed a control diet, 20% gold KF diet, 20% green KF diet or a control diet with a positive control drug (ranitidine) given 1 h prior to aspirin (aspirin +) or water control (aspirin -) treatment. Significant difference *p* < 0.05 between the control diet and kiwifruit diets or positive control is indicated by *, whilst significant difference between the same diet treatment with aspirin treatment compared with no aspirin treatment is indicated by + .

Gene expression results showing significant changes between treatment groups are presented in Figs. 3 (stomach tissue) and 4 (duodenal tissue). The genes with no significant changes in gene expression are presented in the Supplementary Fig. 2.

### Inflammation-related genes

**Figure 4:**
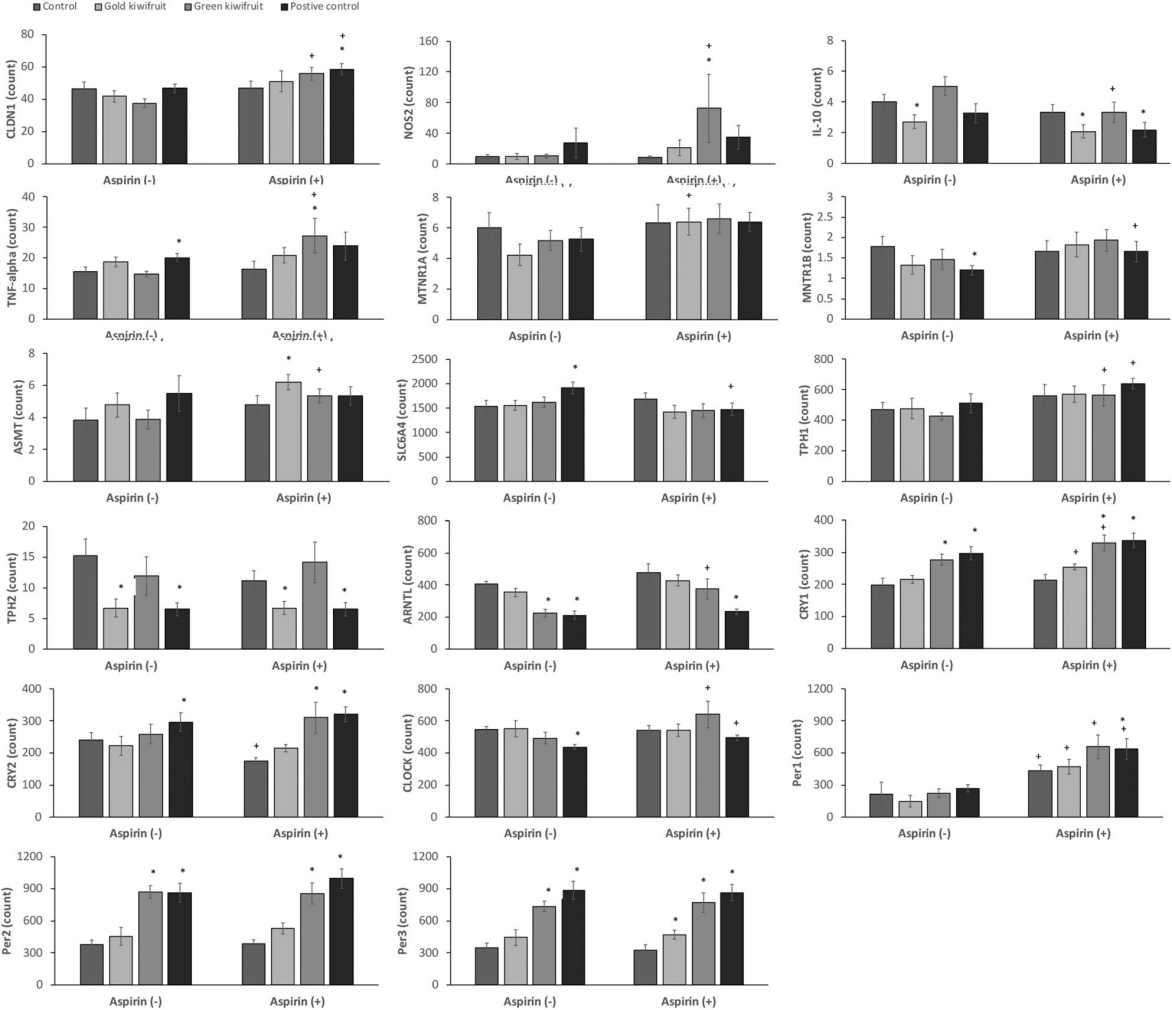
Duodenal gene expression counts normalized to reference genes (n = 8) from rats fed a control diet, 20% gold KF diet, 20% green KF diet or a control diet with a positive control drug (ranitidine) given 1 h prior to aspirin (aspirin +) or water control (aspirin -) treatment. Significant difference *p* < 0.05 between the control diet and kiwifruit diets or positive control is indicated by *, whilst significant difference between the same diet treatment with aspirin treatment compared with no aspirin treatment is indicated by + .

Aspirin treatment did not affect any of the inflammation-related genes (*IL-10*, *TNF-alpha* and *NOS2*) in rats fed the control diet. Dietary treatments had no effects on stomach inflammation-related genes in the non-aspirin treatment groups. However, *NOS2* was increased almost threefold when the positive control drug (ranitidine) was administered. This effect was decreased when aspirin was given, whilst *TNF-alpha* increased following the positive control drug and aspirin combination (Fig. 3). In aspirin-treated rats, *TNF-alpha* and *NOS2* were increased in the duodenum of rats fed the green KF diet (Fig. 4). These genes were not increased by the green KF diet in the absence of aspirin.

### Tight Junction related genes

In the stomach, *OCLN* decreased following aspirin treatment in control and green KF fed rats (Fig. 3). The positive control drug increased *CLDN* 1 and 4 in the stomach of aspirin-treated rats, whilst *CLDN1* was increased in the stomach of rats fed green KF when no aspirin was given. In the duodenum, the combination of green KF and aspirin treatment increased *CLDN1* compared with the green KF diet in non-aspirin-treated rats. The positive control drug increased *CLDN1* in rats given aspirin.

### Genes related to tryptophan metabolism

In the stomach, *TPH1* was decreased in rats fed the green KF diet and aspirin, whilst aspirin treatment in control-fed rats decreased *SLC6A4* (serotonin transporter) and the rats given ranitidine and aspirin had increased *melatonin receptor 1A* (*MNTR1A)* (Fig. 3). In the duodenum, *TPH2* was decreased in non-aspirin-treated rats fed gold KF or ranitidine, whilst ranitidine increased *SLC6A4* in non-aspirin-treated rats, and decreased in aspirin-treated rats (Fig. 4).

### Clock genes

Aspirin treatment decreased *CRY2* in the stomach and increased *PER1* in both stomach and duodenal tissues in all dietary treatment groups. We also observed a significant aspirin effect (*p* = 0.001) with an increase in duodenal *ARNTL* (*Bmal*). Clock genes *PER2* and *3* were strongly positively correlated with *CRY2* (R = 0.74, *PER2* and R = 0.78, *PER3*) in the stomach and *CRY1* (R = 0.81, *PER2* and R = 0.76, *PER3*) in the duodenum. The green KF diet changed the expression of many of the clock genes, such as *ARNTL*, *CRY1*, *CRY2*, *PER*2 and *PER3* when compared with the control diet, whilst the positive control drug ranitidine also effected the expression of these genes. There was no effect of the gold KF diet on these genes.

## Discussion

KF contains compounds that have potential anti-inflammatory effects in vivo, such as MGDG, which was previously observed in the stomach and duodenum of rats fed KF at concentrations that can suppress an inflammatory signaling protein in antigen-stimulated PBMC. However, whether KF has anti-inflammatory effects in preventing gastric damage induced by aspirin in vivo are unknown. In this study, we found that the histological scores were not affected by feeding KF (green or gold) or aspirin administration. There were, however, changes in gene expression, such as an increase in expression of inflammation-related genes *NOS2* and *TNF-alpha* in the duodenum of rats fed the green KF diet. Our study used a higher concentration of aspirin, as used by Thomas et al.^19^ to ensure gastric or duodenal ulcers were present. We used a different aspirin product than in our earlier study^18^, which we speculate is the reason for the lack of visible ulcers in the current study. Whilst our histology analysis failed to produce significant differences between test groups we did observe pro-inflammatory changes at the molecular level.

Aspirin has been found to increase the production of nitric oxide (NO), which can be an indicator of gastric mucosal injury^6^. *NOS2*, which encodes for inducible nitric oxide synthase, has been shown to have a protective role in gastric defense and ulcer repair^20,21^. This was supported in the present study where *NOS2* gene expression was increased 2.8 fold in the stomach of the rats in the positive control ranitidine, no aspirin treatment. In contrast, Liu et al.^22^ reported a decrease in NOS2 activity in the stomach of rats following ranitidine administration, in one study, but in another study an increase in *NOS2* gene expression was observed in the stomach of mice given ranitidine for 14 days followed by ethanol administration to induce gastric damage^23^. As NOS2 activity was not measured in the present study. It is possible that post-transcription modifications may increase *NOS2* gene expression, causing an attenuation of NOS2 activity.

TNF-alpha is known as an inflammatory mediator and has been shown to modulate *NOS2* expression^23^, which can have gastro-protective effects^20,21^. In the present study we found that *NOS2* and *TNF-alpha* expression were increased in the duodenum by 8.3- and 1.7-fold, respectively, in rats receiving the green KF, with aspirin treatment. This may indicate that consumption of green KF potentiates the aspirin-associated pro-inflammatory response, indicating that dietary contraindications should be considered with aspirin consumption. Such a potentiation of aspirin effect was not observed with gold KF, which is much higher in vitamin C compared to green KF. This is interesting considering the protective effect previously demonstrated by vitamin C in aspirin-induced gastric injury^6^.

The two KF cultivars differed in their effects on the expression of inflammation-related genes in the stomach and duodenum, whereby gold KF had no effect and green KF did. The response in the green KF treatment was similar to the transcriptome profile for the rats given ranitidine (positive control). The major difference between nutritional components of green and gold KF cultivars is the higher fiber in green KF and higher vitamin C content in gold KF^1^. There are also likely to be differences in the galactolipid content of green and gold KF, which could account for the differences in gene expression that we observe in our study. KF does contain potential allergens, such as actinidin, which is higher in green KF and this may have influenced duodenal gene expression of TNF-alpha in the rats given green KF diet and aspirin^24^. Another potential mediator of the differences in inflammatory responses between green and gold KF could be the differences in oxalate content^25^. Calcium oxalate has been linked to pro-inflammatory responses^26^. Additionally, dietary polyphenols have been shown to be protective against gastric and duodenal ulcers^27^ and the polyphenol profiles differ between the green and gold KF, with green KF having a higher concentration of many of the polyphenols^28^.

Intestinal tryptophan is metabolized to serotonin and then to melatonin, two important immunomodulatory molecules in the gut. High mucosal concentrations of serotonin are linked to gastrointestinal disorders and inflammation^13,14^. We examined the key genes in serotonin synthesis (*TPH1* and *TPH2*), and reuptake (*SLC6A4*), thereby influencing intestinal serotonin concentrations. We also examined a melatonin receptors (*MNTR1A and MNTR1B*), as a response towards intestinal melatonin content. We observed a decrease in *TPH1* following consumption of green KF in aspirin-treated rats, which may have beneficial effects in inflammatory gut disorders. TPH is a rate limiting enzyme in the biosynthesis of serotonin, which is further converted to melatonin. The TPH1 isoform is the non-neuronal form and expressed in the gut (enterochromaffin (EC) cells) and other peripheral tissues, while TPH2 is expressed in neuronal tissues (e.g. enteroendocrine cells in the duodenum)^29^. *TPH1* expression is upregulated in Crohn’s patients in long-standing remission, whilst *TPH1* knockout mice had reduced severity of colitis induced by dextran sulphate sodium and dinitrobenzene-sulphonic acid in mice^13^, indicating the decrease in *TPH1* observed in our study may be beneficial. Gold KF and the positive control drug, ranitidine, decreased *TPH2* expression in the duodenum of non-aspirin-treated rats, TPH2 affects gastrointestinal motility^14^. In mice lacking TPH2, intestinal transit slowed, whilst gastric emptying accelerated^30^. This corroborated with clinical evidence that eating three gold KF per day to increased spontaneous bowel movements in mildly constipated individuals^31^.

Additionally, we observed a 1.9-fold decrease in expression of the serotonin transporter *SLC6A4* in the stomach following aspirin treatment in control-fed rats, whilst no decreases were observed in rats given the positive control drug or fed KF and given aspirin treatment. In addition, *SLC6A4* increased in the duodenum of rats given the positive control drug without aspirin, indicating a potential role for *SLC6A4* in the protective effect of ranitidine. The serotonin transporter SLC6A4 aids in the removal of mucosal serotonin from the gastrointestinal tract and SLC6A4 is decreased in the epithelia in many inflammatory gastrointestinal disorders^27,28^.

Melatonin and its precursor L-tryptophan play a crucial role in gastro-protection, with Konturek et al.^32^ demonstrating a protective effect of these compounds on aspirin-induced mucosal injury in humans. The main route of action for melatonin in mammals is thought to be via the MNTR1B receptor, since a melatonin receptor blocking drug, luzindole, reversed ulcer healing effects of melatonin in stress-induced rats^33^. Our study showed a 1.6-fold increase in *MNTR1A* in the stomach of rats given the positive control drug with aspirin treatment compared with no aspirin treatment. Previous research has also shown that melatonin can increase *NOS2* gene expression and this may contribute to the gastro-protective effects of melatonin^33^. In our study, we saw no correlation with melatonin receptor expression and *NOS2* expression, despite showing that the positive control drug increased *NOS2* in non-aspirin-treated rats.

Tight junction expression in the stomach is less well understood than in intestinal tissues, particularly when observing changes in the architecture of stomach tight junctions during inflammation and infection^34^. In the present study, the stomach *CLDN1* gene expression increased 2.5-fold following the green KF diet in the absence of aspirin and twofold in the ranitidine with aspirin treatment. An increase in *CLDN1* in the intestine, has been linked to inflammatory disorders^35^ and Poritz et al.^36^ showed CLDN1 increased and OCLN decreased in a rat ileal cell line treated with pro-inflammatory TNF-alpha. We observed an increase in duodenal *TNF-alpha* that correlated with the effect on *CLDN1* in the green KF group, indicating the green KF may induce an inflammatory response potentially irritating the gastric membranes. We observed a lower expression of stomach *OCLN* in control-fed or green KF-fed rats with aspirin treatment. This result is supported by the study of Thakre-Nighot et al. (2016) who observed a decrease in occludin expression in a gastric cell line and mouse gastric mucosa, with no changes in *CLDN 1* or *4* expression, following exposure to the NSAID indomethacin^37^. The positive control drug ranitidine increased stomach *CLDN4* expression by 1.8-fold compared with the control in the aspirin-treated rats. CLDN4 has previously been shown to be down-regulated in intestinal inflammation disorders^35,38^, indicating this may be a beneficial effect of the ranitidine on barrier integrity.

Clock genes play a critical role in regulating various genes^39^, such as those involved in immunity. In the gastrointestinal tract, the molecular clock can be synchronized by timed feeding, independently of the central circadian clock^40^. Changes in the expression of the clock genes can alter the phase or amplitude of these genes in a 24 h cycle ultimately affecting clock-dependent genes, resulting in a regulation of 10% host genome^17^. Aspirin treatment in our study reduced stomach *CRY2* expression and increased *PER1* in both stomach and duodenal tissues and across all treatment groups, indicating a mechanism in which aspirin maybe affecting other genes in our panel. PER1 is released by the gastric oxyntic gland cells in a circadian fashion, and is correlated with central clock rhythms of orexin^41^, which has been linked with a defense response and arterial blood pressure^42^. Previous research on aspirin showed that administration at night and not in the morning could reduce hypertension in humans^43^. However, a study in mice showed that aspirin had no effect on clock genes in the kidney, heart, adrenal and aorta tissues irrespective of when it was given (night or morning)^44^. This is the *first study* to show that aspirin affects the expression of clock genes in the stomach and duodenum and this may have implications for the timing of dosing for maximum beneficial effect. In addition, *PER 2* and *3* were strongly positively correlated with *CRY2* in the stomach and *CRY1* in the duodenum, indicating different synchronization between tissues. Feeding with green KF changed the expression of the many of clock genes, whilst gold KF did not. Green KF affected many more genes tested in our study compared with gold KF, particularly when in combination with aspirin. This may be partly mediated by changes in clock gene expression, which were also affected more by the green KF diet in comparison to the gold KF diet.

Histological evaluation and scoring revealed that a 400 mg/kg aspirin dose, given to rats as the active ingredient in the product Aspro Clear, was ineffective in causing mucosal inflammatory cell infiltration or ulcers in this study. We did, however, develop a new histological scoring system to measure inflammatory cells the number of inflammatory cells in the mucosa layers, which may indicate ulcer formation following aspirin treatment^19^. Methodology for scoring rat stomach inflammation is absent from the literature, other than that of Eaton et al*.*, who quantified histological lesions in gastric epithelium of mice infected with *Helicobacter pylori*^45^. However, the features seen in the gastric tissue were quite different from the rat gastric tissue of the rats in this study. For example, we observed inflammatory cells infiltrating aspirin-treated and untreated gastric epithelium, with no lesions or clusters of neutrophils, as observed in the Eaton et al*.* study^45^. Whilst, our aspirin dose was ineffective in causing enhanced mucosal inflammatory cell infiltration or ulcers, this scoring method could be utilized by researchers assessing localized inflammation in the stomach. We also recommend the use of pure aspirin for animal studies of gastric inflammation, rather than branded formulations as used in our study as we speculate that the added ingredients, such as sodium bicarbonate and citric acid, in Aspro Clear protect against ulcer formation.

In summary, the results of this study show that whilst aspirin treatment used in this study did not result in stomach ulcers or an increase in the number of inflammatory cells in the stomach tissue, it did influence the expression of genes involved in inflammation, hormone-mediated signaling and circadian rhythm. Most notable are the effects of aspirin on clock genes that have not been described previously and may have a significant impact on timing of delivery of aspirin to minimize adverse effects and/or maximize the benefits. We also demonstrated that gold and green KF diets have some opposing effects on some genes related to inflammation and gastro-protective effects, which require further investigation as to what components might mediate these responses.

## Materials and methods

### Kiwifruit

Green- and gold-fleshed KF were supplied by Zespri International, Tauranga, New Zealand. The KF were peeled, blended to a homogenous mix and immediately frozen in 500 g measures. The KF diets were prepared as shown in Supplementary Table S1 and frozen in daily batches. The concentration of homogenized fresh KF in the experimental diets was 20%. This dose is equivalent to a human consuming four fresh whole KF per day: four KF would be approximately 14% of daily food intake assuming one KF weighs 75 g and the mean human food intake is 2.09 kg/d^46^.

### Experimental design

**Figure 5:**
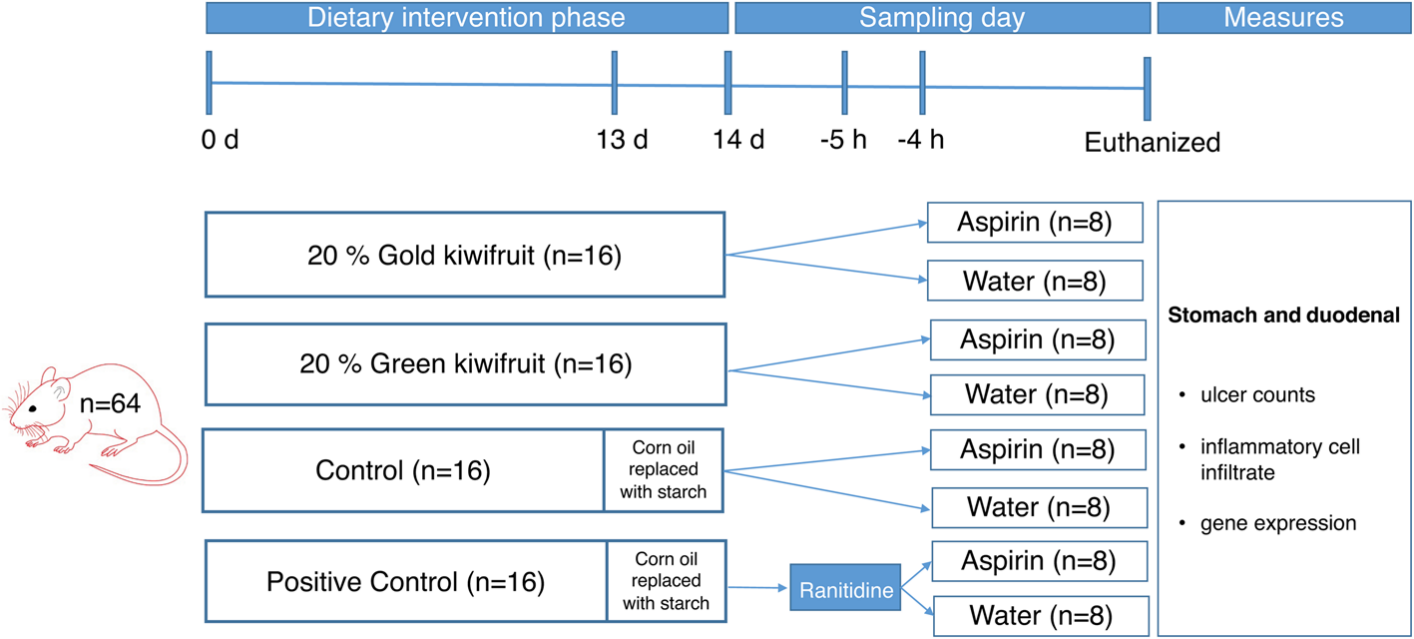
Illustration overview of the experimental design.

Sixty-four weanling male Sprague–Dawley rats (45–50 g live weight) were housed in shoebox cages in family groups in a room maintained at a temperature of 22 ± 1 °C, humidity of 60 ± 5%, air exchange of 12 times/h, and with a 12-h light/dark cycle, with lights on at 0,700 h. An overview of the experimental design is shown in Fig. 5. This study was approved by AgResearch Grasslands Animal Ethics Committee (Palmerston North, New Zealand), according to the Animal Welfare Act 1999, New Zealand. All the rats were fed a commercial diet ad libitum until they reached 160–210 g live weight. They were moved to individual cages and randomly allocated to the experimental treatments. There were eight experimental treatments (n = 8): control diet with aspirin; control diet without aspirin; gold kiwifruit (gold KF) diet with aspirin; gold KF diet without aspirin; green kiwifruit (green KF) diet with aspirin; green KF without aspirin: control diet and ranitidine (positive control) with aspirin; and control diet and ranitidine without aspirin. The positive control rats received a single dose of ranitidine on the last day of the study 1 h prior to the aspirin administration. The ranitidine (50 mg/kg bodyweight) was dissolved in water (trade name Zantac, containing ranitidine hydrochloride, 300 mg per tablet) and administered by oral gavage^47^. The rats were fed the experimental diets ad libitum throughout the 14-day study (Supplementary Table S1). Rat weights and food intakes were recorded weekly during the 14-day trial. On the final day, the rats were fed their experimental diets with the corn oil removed (replaced by starch) to reduce the quantity of dietary α-linoleic acid not from kiwifruit entering the gastrointestinal tract 24 h prior to aspirin administration. At the end of the 14-day feeding period, all rats were fasted overnight (16 h) and then given 400 mg/kg aspirin or water (control) via oral gavage^19^. The aspirin used was Aspro Clear (Bayer plc, Newbury, UK), which comprises: aspirin 300 mg, sodium bicarbonate 508 mg, citric acid anhydrous 362 mg per tablet). The animals were euthanized by CO_2_ overdose 4 h after aspirin or water administration. A blood sample was taken from each rat via cardiac puncture and placed in a tube with ethylenediaminetetraacetic acid as an anticoagulant for the preparation of plasma. The stomach was immediately removed, opened along the greater curvature, rinsed with cold phosphate buffered saline (0.01 M) and inspected for macroscopic damage. A sample of gastric glandular epithelium was removed and fixed in 10% formalin for histological analysis. A second sample of gastric glandular epithelium was stored in RNAlater (Life Science Research Technologies, ThermoFisher Scientific, New Zealand) at -80 °C. Sampling was staggered over 4 d, with 16 rats being euthanised between 12 and 4 pm per day. Dosing of aspirin was staggered to ensure each rat received aspirin 4 h prior to euthanasia.

### Histological evaluation of gastric mucosal inflammation

The ulcers in the whole stomach tissue of each rat were examined under a dissecting microscope with a square grid at 6 × magnification (Wild M5A, Wild Heerbrugg, Switzerland). As described by Ribeiro et al.^48^, the ulcers were classified as level I ulcer area < 1 mm^2^, level II ulcer area 1–3 mm^2^, level III ulcer area > 3 mm^2^. On the basis of the ulcer classification, the following parameters were determined: (i) ulcer area (mm^2^); (ii) ulcer lesion index (ULI) = 1 × (number of ulcers at level I) + 2 × (number of ulcers at level II) + 3 × (number of ulcers at level III); and (iii) curative ratio (%) = 100 (ULI treated/ULI control × 100). The ulcer area was calculated by using the sum of the areas of ulcers for each stomach as described by Kauffman and Grossman^49^.

A section was excised from the gastric glandular epithelium at a region located 2 mm below the limiting ridge that separates the forestomach from the glandular epithelium along the greater curvature of the stomach of each rat^50^. Samples were fixed in 10% neutral buffered formalin, processed overnight using sequential dehydration and clearing steps (70%, 95%, 100%, 100% ethanol, 50/50 ethanol/xylene mix and 100% xylene × 3 changes, respectively) and then embedded in paraffin. For microscopic examination, 5 μm thick sections (two per rat) were generated and stained with haematoxylin and eosin.

A histological examination method for evaluating gastric injury in rats was developed for this study. The limiting ridge, a junction between the forestomach and the glandular stomach represented by a fold of the forestomach mucosa, was located using a Zeiss Axio Imager Z2 microscope (Carl Zeiss AG, Oberkochen, Germany) (magnification 40 x). Subsequently, seven images (magnification 400 x) were taken per rat of the glandular stomach (fundus) by following the interface of the mucosa and submucosa immediately adjacent to the limiting ridge. Inflammatory cells were identified by a segmented nucleus with a reddish granulated cytoplasm and were counted in each image by a blinded investigator using the ZEN 2.6 (blue edition) software. A mean inflammatory cell count per rat was determined by calculating the mean of the counts from the seven images. A Poisson generalized linear model was used to compare differences between treatment groups.

### RNA extraction from stomach and duodenal tissue

Stomach and duodenal tissue were thawed and blotted to remove excess RNAlater. A section of the tissue (15–25 mg) was homogenized at maximum speed for 20 s with an OMNI THQ TM homogenizer (OMNI International, USA) in 700 µL RLT buffer (Qiagen, Germany). The samples were then centrifuged at 17,000 g for 5 min at room temperature and the supernatant was transferred to a new tube. An equal volume of 70% molecular grade ethanol was added to the supernatant and transferred to a Qiagen RNeasy MINI column (Qiagen, Germany). RNA was extracted as per the manufacturer’s protocol (RNeasy mini handbook, Fourth edition, June 2012) into 60 µL of water for gene expression of 24 genes (Supplementary Table S2) by the Counter Analysis System (NanoString Technologies, USA). RNA quality and quantity were assessed by NanoDrop Spectrophotometer ND-1000 (NanoDrop Technologies, Inc., USA).

### Gene expression analysis – NanoString nCounter Analysis System

RNA samples (1,450 ng stomach and 800 ng duodenum) were analyzed using the nCounter Plexset reagents (NanoString, Seattle, USA) following the manufacturer’s instructions. Target sequences were designed by NanoString Technologies, Inc. and ordered from Integrated DNA Technologies, Inc., Iowa, USA (Supplementary Table S2). Samples were prepared as previously described in Bentley-Hewitt et al.^51^. Initially sample were spiked with six different internal control probes at concentrations ranging from 128 fM to 0.125 fM in fourfold dilution steps. Eight positive controls were used to determine the linearity of the assays and were used for normalization. Eight negative probes were used to control for carryover of reporter probes as no RNA target was included for these probes. RNA samples were incubated for 24 h at 65 °C in hybridization buffer containing the CodeSet, which consisted of reporter and capture probes and together with the target RNA formed a tripartite complex. After hybridization, the complex was bound by its biotin-labeled capture probe on a streptavidin-coated glass slide and was stretched within an electric field. Hybridized samples were processed using the robotic Prep Station (High Sensitivity Protocol, 3 h per 12-sample cartridge) and data acquisition was performed by using the GEN2 Digital Analyzer, with the ‘Max’ Field of View setting (555 images per sample; 5-h scan per cartridge). Raw counts were normalized using the positive controls, and target genes were normalized to the internal reference genes; Glucose-6-phosphatase catalytic subunit (*G6PC*), RNA polymerase I subunit B (*POLR1B*), TATA box binding protein (*TBP*), Hypoxanthine Phosphoribosyltransferase 1 (*HPRT1*), Succinate Dehydrogenase Complex Flavoprotein Subunit a (*SDHA*).

### Statistical analysis

Poisson generalized linear models with factors for aspirin (Yes/No) and diet (Control, Gold KF, Green KF), plus the interaction between diet and aspirin treatment, were used to compare ulcer scores, inflammatory cell counts and gene expression. The residual deviance was compared with the residual degrees of freedom and, if significantly higher, an over-dispersion factor was estimated and used in the final analysis. Pairwise likelihood ratio tests were used to test whether pairs of treatments has significantly different means. Analysis of variance (ANOVA) with Aspirin and Diet as factors, plus the interaction, was used to assess food intake and weight gain. Principal components analysis (PCA) on the variance–covariance matrix of the logs of gene expression was used to summarize the data from the stomach and from the duodenum (PCA on the variance–covariance matrix of log-transformed data identifies the largest sets of fold changes in the data). Pearson’s correlations were also calculated between the log-transformed expressions for different genes. The analysis was carried out using GenStat 14th edition (VSN International, Hemel Hempstead, UK).

### Ethical approval

All procedures performed in studies involving animals were in accordance with ethical standards of the institution at which the studies were conducted.

## Supplementary information

Supplementary information
