# Supplementary material for: Influence of kiwifruit on gastric and duodenal inflammation-related gene expression in aspirin-induced gastric mucosal damage in rats
Source: Sci Rep. 2020 Aug 3;10:13055. doi: 10.1038/s41598-020-70006-0 (PMC7400567; doi:10.1038/s41598-020-70006-0)

Influence of kiwifruit on gastric and duodenal inflammation-related gene expression in aspirin-induced gastric mucosal damage in rats

Kerry L. Bentley-Hewitt^1,*^, Matthew Perrott^2^ Christine A. Butts^1^, Duncan I. Hedderley^1^, Halina M. Stoklosinski^1^, Shanthi G.Parkar^1^

^1^The New Zealand Institute for Plant and Food Research Limited, Private Bag 11600, Palmerston North 4442, New Zealand.

^2^School of Veterinary Science, Massey University, Palmerston North, New Zealand.

^*^Corresponding author. Tel.: +64 6 355 6215; fax: +64 9 925 7001.

**Supplementary Materials**

**Supplementary tables**

**S1.** Composition of the experimental diets (g/kg)

| Ingredient | Control | Green kiwifruit | Gold kiwifruit |
| --- | --- | --- | --- |
| Lactic casein^a^ | 140 | 140 | 140 |
| Vitamin mixture^b^ | 50 | 50 | 50 |
| Mineral mixture^c^ | 50 | 50 | 50 |
| Corn oil^d^ | 50 | 50 | 50 |
| Starch^e^ | 460 | 460 | 460 |
| Cellulose^f^ | 50 | 50 | 50 |
| Green kiwifruit |  | 200 |  |
| Gold kiwifruit |  |  | 200 |
| 10% glucose solution^g^ | 200 |  |  |

^a^Alacid80mesh, New Zealand Milk Products, Wellington, New Zealand. ^b^ Mixture contains the following components: (mg/kg diet) – retinol acetate 5.0, DL-α-tocopheryl acetate 100.0, menadione 3.0, thiamine hydrochloride 5.0, riboflavin 7.0, pyridoxine hydrochloride 8.0, D-pantothenic acid 20.0, folic acid 2.0, nicotinic acid 20.0, D-biotin 1.0, myo-inositol 200.0 and choline chloride 1500.0; (μg/kg diet) – ergocalciferol 25.0 and cyanocobalamin 50.0.

^c^Mixture contains the following components: (g/kg diet) – Ca 6.29, Cl 7.79, Mg 1.06, P 4.86, K 5.24 and Na 1.97; (mg/kg diet) – Cr 1.97, Cu 10.7, Fe 424.0, Mn 78.0 and Zn 48.2; (μg/kg diet) – Co 29.0, I 151.0, Mo 152.0 and Se 151.0. ^d^Davis Trading Company, Palmerston North, New Zealand. ^e^Wheat starch, Grain Foods, Tamworth, Australia. ^f^Ceolus PH-102, Asahi Kasei Chemicals Corporation, Tokyo, Japan. ^g^ Sigma-Aldrich, New Zealand.

**S2.** Gene targets analyzed by NanoString Technologies

| **Gene Name** | **GenBank accession number** | **Target region** | **Target Sequence** |
| --- | --- | --- | --- |
| Glucose-6-phosphatase catalytic subunit (*G6PC*) | NM_013098.2 | 155-254 | TTGTCTTGGTGTCTGTGATCGCTGACCTCAGGAACGCCTTCTATGTCCTCTTTCCCATCTGGTTCCACATTCAAGAGACTGTGGGCATCAATCTCCTCTG |
| RNA polymerase I subunit B (*POLR1B*) | NM_031773.1 | 2786-2885 | ATGCCGGACATTCTGTTTAATCCTCATGGGTTTCCCTCCCGTATGACCATAGGTATGTTAATCGAGAGTATGGCTGGGAAGTCAGCAGCTTTGCATGGTC |
| TATA box binding protein (*TBP*) | NM_001004198.1 | 151-250 | TGTCCCCACATCACTGTTTCATGGTGCGTGACGATAACCCAGAAAGTCGAAGACGTTTCTAAGGAGATAAGAGGATGCTCTAGGAAAAATCTGAGTACCG |
| Hypoxanthine Phosphoribosyltransferase 1 (*HPRT1*) | NM_012583.2 | 21-120 | AGCTTCCTCCTCAGACCGCTTTTCCCGCGAGCCGACCGGTTCTGTCATGTCGACCCTCAGTCCCAGCGTCGTGATTAGTGATGATGAACCAGGTTATGAC |
| Succinate Dehydrogenase Complex Flavoprotein Subunit A (*SDHA*) | NM_130428.1 | 1461-1560 | CCTCCGATTAAGGCAAATGCTGGAGAAGAGTCGGTTATGAATCTTGACAAGTTGAGATTTGCTGATGGAAGTGTAAGAACATCAGAGCTGCGCCTCAGCA |
| Period 1 (*PER1*) | NM_001034125.1 | 706-805 | CAGGATACCTTCTCGGTGGCTGTGTCCTTCCTGACAGGCCGGATTGTCTATATTTCGGAGCAGGCAGGTGTCTTGCTGCGTTGCAAACGGGATGTGTTTC |
| Period 2 (*PER2*) | NM_031678.1 | 5581-5680 | GTAAATAAGCTCTCAGAGTTTGTGCGATGATTTGTGAGCCTTGCCGGACAAGCGGTTTGTTCATGCGCAAACCAAACGTACCTTCACCCAGTGCAATATA |
| Period 3 (*PER3*) | NM_023978.2 | 406-505 | TTTTCGTTTCTGTCTGGAAGGCTGGTGCACATTTCTGAACAGGCTGCTTGGATCCTGAATTCTAAGAAAGGTTTCCTCAAGAGCTTGCACTTCGTCGACC |
| Circadian Locomotor Output Cycles Kaput (*CLOCK*) | NM_021856.1 | 1421-1520 | CACAGCGGAGGTCATCCTTCAGTAGTCAGTCCATAAACTCCCAGTCAGTTGGTTCATCATTAACACAGCCAGCGATGTCTCAAGCTGCAAATTTACCAAT |
| Aryl hydrocarbon receptor nuclear translocator-like protein 1 (*ARNTL*) | NM_024362.2 | 301-400 | AAAATGACTGTCTAGGTGGAGGATTTTGGAAGAAGTTGACTGCCTGGAAGAAAGTTACAAAACATGAAAGTCGCTTTGAGGTGACCAGCAAGTACAGTGG |
| Cryptochrome Circadian Regulator 1 (*CRY1*) | NM_198750.2 | 681-780 | ATCAACAGGTGGCGATTTTTGCTTCAGTGTCTTGAGGACCTTGATGCCAATCTACGAAAGTTAAATTCTCGCCTGTTTGTGATCCGGGGACAGCCAGCTG |
| Cryptochrome Circadian Regulator 2 (*CRY2*) | NM_133405.1 | 1393-1492 | TATATCTATGAGCCCTGGAATGCTCCCGAGTCGGTTCAGAAGGCCGCTAATTGCATCATTGGCGTGGACTACCCACGGCCCATCGTTAACCACGCAGAGA |
| Melatonin receptor type 1A (*MTNR1A*) | NM_053676.2 | 1237-1336 | TAATAATTTAATAAAGGTGGACTCTGTTTAAAAAAGCCAGTGGTGCCAGCAGGTTACGCTCGCTGGTTGGGGTCTTACTGCTTTCTCTTGTCTAGAAATC |
| Melatonin receptor type 1B (M*TNR1B*) | NM_001100641.1 | 599-698 | AGCCTCATCTGGCTTCTCACTCTGGTGGCCTTGGTGCCCAATTTCTTTGTGGGGTCTCTAGAATATGACCCGCGAATCTATTCCTGCACCTTCATCCAGA |
| N-Acetylserotonin O-methyltransferase (*ASMT*) | NM_144759.2 | 1405-1504 | ACCAAAACCTGTGACTTGGGATAGGAAGTGACATCATGGGTGGGAATTTATGACTTCTGGACAGTTACATTACAGCTGAGAGTCAGTGAGTTTTAGGCAG |
| Tumour necrosis factor alpha (*TNF-alpha*) | NM_012675.2 | 306-405 | GGTGATCGGTCCCAACAAGGAGGAGAAGTTCCCAAATGGGCTCCCTCTCATCAGTTCCATGGCCCAGACCCTCACACTCAGATCATCTTCTCAAAACTCG |
| Interleukin 10 (*IL10*) | NM_012854.2 | 186-285 | ACAACATACTGCTGACAGATTCCTTACTGCAGGACTTTAAGGGTTACTTGGGTTGCCAAGCCTTGTCAGAAATGATCAAGTTTTACCTGGTAGAAGTGAT |
| Nitric Oxide Synthase 2 (*NOS2*) | NM_012611.2 | 21-120 | ACGGGACACAGTGTCGCTGGTTTGAAACTTCTCAGCCACCTTGGTGAGGGGACTGGACTTTTAGAGACGCTTCTGAGGTTCCTCAGGCTTGGGTCTTGTT |
| Tryptophan hydroxylase 2 (*TPH2*) | NM_173839.2 | 1581-1680 | TTGTCAGCACGAGCTCTTGGGGGCTTAGCAACAATGCAGTCAATGTTATCCAACATCAACAACTTTCTGTGTCATGGCTGGCTAGTAAGCATGCAATTCC |
| Tryptophan hydroxylase 1 (*TPH1*) | NM_001100634.2 | 2203-2302 | AGGAGAGCAGTTTGTATCAGATTGTAACTCTGTGAGGGCTGTAAGGATTGGAGCACGTGTGGTGTCAGCAGTGTAGCCTTGGCATAAGCAGTTGTATAAA |
| Claudin-1 (*CLDN1*) | NM_031699.2 | 2741-2840 | AAGCAAATGCAATGGCGGAAAGTGACAGTGTATGGATATTCTAAAAGCATAAAGCGTGCTCTTTGTGAAGTGATACTTTCAAAGTCTAATGGAAGCTGGC |
| Occludin (*OCLN*) | NM_031329.2 | 91-190 | TCTTGGGAGCCTTGACATCTTGTTCATCATAAAGATCAGGTGACCAGTGACATCAGCCATGTCTGTGAGGCCTTTTGAGAGTCCACCTCCTTACAGGCCG |
| Claudin-4 (*CLDN4*) | NM_001012022.1 | 1167-1266 | AGTGGATGTCCTGGTCATGTTAGCAAACATTCACTGCCCTTTCTCAGTGCCCTCGCTCTCTCGCCTCCACGTTACTCCCGCGCTACTCTTGCCGTTTCTC |
| Solute Carrier Family 6 Member 4 (*SLC6A4*) | NM_013034.3 | 587-686 | CATTGGTTACGCCATCTGCATCATCGCCTTTTACATCGCCTCCTACTACAACACCATCATAGCCTGGGCGCTCTACTACCTCATCTCCTCCCTCACGGAC |

**S3.** Body weight and food intake of rats (n=8) fed experimental diets with and without aspirin treatment.

| **Treatment groups** | **Weight gain**  **(g/14 days)** | **Food Intake^1^**  **(g/day)** |
| --- | --- | --- |
| Control | 97.3 | 28.1 |
| Control + Aspirin | 95.4 | 26.9 |
| Gold KF | 99 | 27.6 |
| Gold KF + Aspirin | 97.3 | 27.5 |
| Green KF | 98.7 | 28.1 |
| Green KF + Aspirin | 98.5 | 28.7 |
| Ranitidine | 100 | 29.1 |
| Ranitidine + Aspirin | 93.5 | 28.1 |
| *P* values |  |  |
| Diet group (3 df) | 0.965 | 0.615 |
| Aspirin treatment (1 df) | 0.481 | 0.586 |
| Diet x Aspirin (3 df) | 0.935 | 0.809 |

^1^Food intake per day during the experiment diet feeding period of 2 weeks

df - Degrees of freedom

**Supplementary figures**

**F1.** Inflammatory cell counts in stomach tissues shown as a mean count (n=8 rats, n=7 x 40 objective images per rat shown as a total count per image (example images are shown in Figure 1). No significant differences were observed.

**F2.** Additional stomach (a) and duodenal (b) gene expression counts normalized to reference genes (n=8) from rats fed a control diet, 20 % gold KF diet, 20 % green KF diet or a control diet with a positive control drug (Ranitidine) given 1 hour prior to aspirin (aspirin +) or water control (aspirin -) treatment.


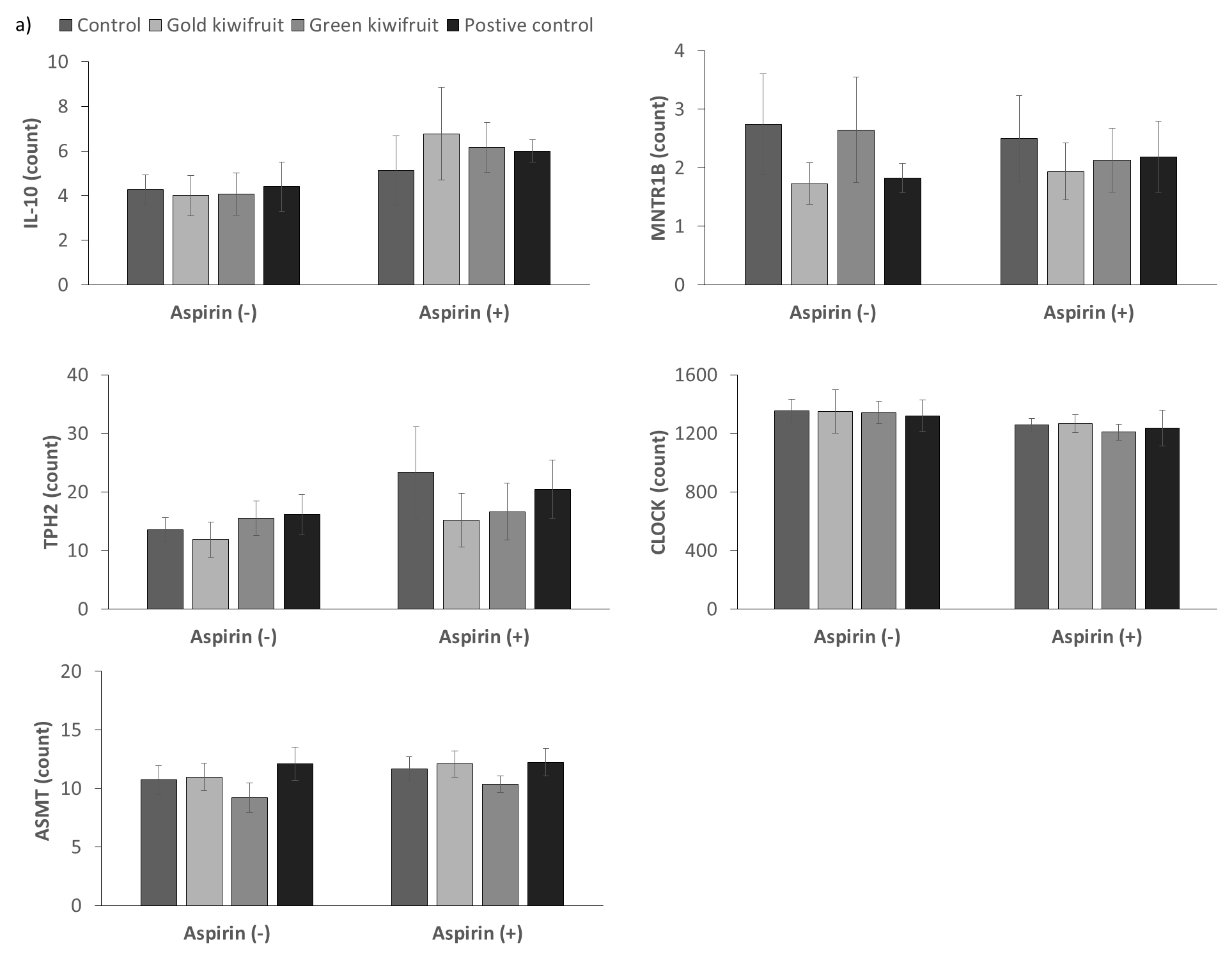

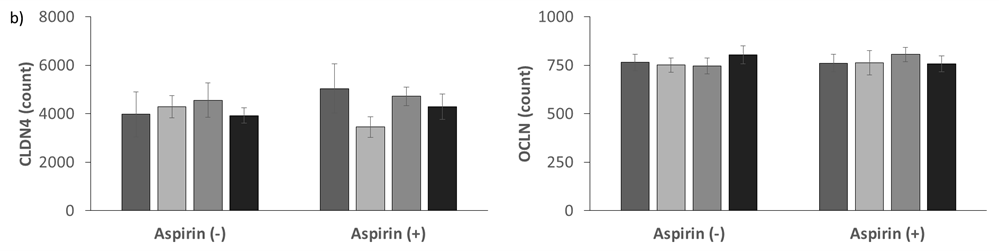

Supplement: Supplementary file 1 — Supplementary information [file 41598_2020_70006_MOESM1_ESM.docx]
